# The Amyloid Fibril-Forming β-Sheet Regions of Amyloid β and α-Synuclein Preferentially Interact with the Molecular Chaperone 14-3-3ζ

**DOI:** 10.3390/molecules26206120

**Published:** 2021-10-11

**Authors:** Danielle M. Williams, David C. Thorn, Christopher M. Dobson, Sarah Meehan, Sophie E. Jackson, Joanna M. Woodcock, John A. Carver

**Affiliations:** 1 Department of Chemistry, School of Physical Sciences, The University of Adelaide, Adelaide, SA 5001, Australia; dwilliams@biocina.com; 2 Research School of Chemistry, The Australian National University, Acton, ACT 2601, Australia; dr.david.thorn@gmail.com; 3 Yusuf Hamied Department of Chemistry, University of Cambridge, Lensfield Road, Cambridge CB2 1EW, UK; sarah.meehan.cam@gmail.com (S.M.); sej13@cam.ac.uk (S.E.J.); 4 Centre for Cancer Biology (An Alliance between SA Pathology and the University of South Australia), Adelaide, SA 5000, Australia; Joanna.Woodcock@sa.gov.au

**Keywords:** 14-3-3 proteins, molecular chaperone, amyloid β, α-synuclein, NMR spectroscopy, amyloid fibril

## Abstract

14-3-3 proteins are abundant, intramolecular proteins that play a pivotal role in cellular signal transduction by interacting with phosphorylated ligands. In addition, they are molecular chaperones that prevent protein unfolding and aggregation under cellular stress conditions in a similar manner to the unrelated small heat-shock proteins. In vivo, amyloid β (Aβ) and α-synuclein (α-syn) form amyloid fibrils in Alzheimer’s and Parkinson’s diseases, respectively, a process that is intimately linked to the diseases’ progression. The 14-3-3ζ isoform potently inhibited in vitro fibril formation of the 40-amino acid form of Aβ (Aβ_40_) but had little effect on α-syn aggregation. Solution-phase NMR spectroscopy of ^15^N-labeled Aβ_40_ and A53T α-syn determined that unlabeled 14-3-3ζ interacted preferentially with hydrophobic regions of Aβ_40_ (L11-H21 and G29-V40) and α-syn (V3-K10 and V40-K60). In both proteins, these regions adopt β-strands within the core of the amyloid fibrils prepared in vitro as well as those isolated from the inclusions of diseased individuals. The interaction with 14-3-3ζ is transient and occurs at the early stages of the fibrillar aggregation pathway to maintain the native, monomeric, and unfolded structure of Aβ_40_ and α-syn. The N-terminal regions of α-syn interacting with 14-3-3ζ correspond with those that interact with other molecular chaperones as monitored by in-cell NMR spectroscopy.

## 1. Introduction

Protein aggregation is a characteristic of many diseases, the majority of which are age-related and neurological. Common examples of protein aggregation diseases (also known as protein misfolding or protein conformational diseases) include Alzheimer’s (AD), Parkinson’s (PD), Huntington’s, and Creutzfeldt–Jakob [1,2]. The protein aggregates or deposits associated with these diseases contain a predominant peptide or protein that, in the majority of cases, adopts an amyloid fibrillar form. Amyloid fibrils are a highly stable, aggregated proteinaceous state with the polypeptide arranged mainly in a cross β-sheet conformation that results in an extended, overall fibrillar structure up to micrometers in length [1,2]. The conversion of a protein from its native, functional state to an amyloid fibril is a multi-step process that usually involves a nucleation-dependent mechanism that has various intermediate states, including the formation of prefibrillar oligomers that act as nuclei to sequester and convert natively structured proteins into the fibrillar form. The prefibrillar oligomers are proposed to be the entities that cause cell toxicity and, hence, are intimately involved in the disease processes [1,2].

Within and outside cells, protein levels and their conformations are maintained within a narrow regime to minimize the possibility of protein unfolding, misfolding, and aggregation. The general term for this process is proteostasis, a portmanteau of protein and homeostasis [3]. The action of molecular chaperone proteins in stabilizing the conformation, stoichiometry, and interactions of other proteins is one of the major means of maintaining cellular proteostasis. Under stress conditions such as elevated temperature, the principal intracellular chaperones that function to prevent protein unfolding and aggregation are the small heat-shock proteins (sHsps) [4]. Their levels are upregulated many-fold under such conditions and with aging [4] and in diseases associated with protein aggregation [5,6]. Overall, the ATP-independent action of sHsps is crucially important in cellular well-being under normal and stress conditions.

In addition to sHsps, other proteins have an sHsp-like chaperone ability that may supplement or complement that of sHsps. In this context, intracellular 14-3-3 proteins exhibit chaperone action against a variety of unfolding proteins under stress conditions such as elevated temperature [7,8,9,10]. They are present at high levels in the brain. In humans, there are seven closely related 14-3-3 proteins. The principal role of 14-3-3 proteins is their involvement in cellular signal transduction processes via their interaction with phosphorylated substrate proteins. As such, they function as adapters and participate in a variety of cellular pathways, including apoptosis, transcription and the stress response [9]. In addition to their intracellular presence, 14-3-3 proteins are found extracellularly, for example in exosomes and in cerebrospinal fluid of people with neurodegenerative diseases such as Creutzfeldt–Jakob disease, AD, and multiple sclerosis [11]. 14-3-3 proteins are dimers composed of subunits of ~28 kDa in mass. Each subunit adopts a predominantly α-helical conformation with nine α-helices. The dimer is arranged in a double cup-like shape with two amphipathic binding grooves where phosphorylated ligands bind. The dimer interface is provided by an interaction between N-terminal helices of each subunit.

The two major neurological diseases associated with protein aggregation and deposition are AD and PD [1,2]. AD is characterized by the extracellular deposition of plaques containing mainly variants of the amyloid beta peptide (Aβ) in an amyloid fibrillar form, along with intracellular neurofibrillary tangles containing mainly the tau protein. The principal Aβ peptides are 40 and 42 amino acids in length. In PD, Lewy body deposits are the defining morphological intracellular feature; they are mainly comprised of the protein α-synuclein (α-syn), also deposited as amyloid fibrils. In AD and PD, other proteins are associated with these deposits, including sHsps and 14-3-3 proteins [9,12,13]. Their presence may arise from the utilization by cells of their chaperone ability as an attempt to prevent the aggregation of Aβ and α-syn to form amyloid fibrils. 

In this study, we report on an NMR spectroscopic and biophysical analysis of the interaction of 14-3-3ζ, a major 14-3-3 isoform, with Aβ, and a similar interaction between 14-3-3ζ and A53T α-syn, a mutant of α-syn associated with familial PD that aggregates more rapidly than the wild type (WT) protein. The findings have implications for the in vivo association of these species and their involvement in AD, PD, and other related diseases of protein aggregation where amyloid fibrillar aggregation occurs.

## 2. Results and Discussion

### 2.1. Interaction of 14-3-3ζ with Amyloid β Peptides

**Figure 1:**
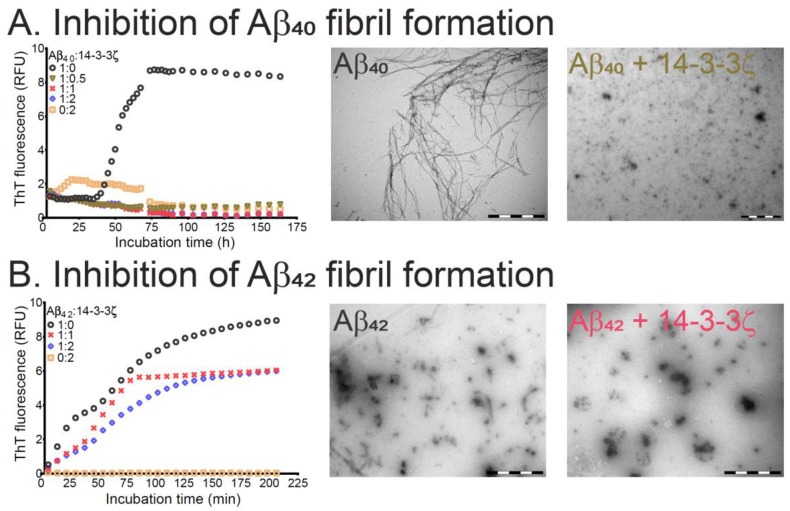
The molecular chaperone ability of 14-3-3ζ to inhibit amyloid fibril formation of Aβ_40_ and Aβ_42_. Amyloid fibril formation, as monitored by ThT fluorescence, of 15 μM Aβ_40_ (**A**) and Aβ_42_ (**B**) at 37 °C in the absence (black circles) and presence of 14-3-3ζ at 1.0:0.5 (brown triangles; Aβ_40_ only), 1:1 (red crosses), and 1:2 (blue diamonds) molar ratios of Aβ_40_/Aβ_42_:14-3-3ζ. Also shown is 14-3-3ζ incubated alone at 30 µM (beige squares). On the right of part (**A**) are TEM images of Aβ_40_ in the absence and presence of 14-3-3ζ at a 1.0:0.5 molar ratio of Aβ_40_:14-3-3ζ. Samples for TEM imaging were taken upon completion of the aggregation assay after 150 h of incubation. On the right of part (**B**) are TEM images of Aβ_42_ in the absence and presence of 14-3-3ζ at an equimolar ratio. Each image was acquired from the samples after 200 min. of incubation. Scale bars in all images represent 1 μm.

Thioflavin T (ThT) is a dye whose fluorescence increases markedly upon binding to the β-sheet regions of amyloid fibrils, a phenomenon that is routinely utilized to monitor amyloid fibril formation in peptides and proteins [14]. Figure 1 shows the ThT fluorescence profiles for the 40- and 42-amino acid forms of Aβ (Aβ_40_ and Aβ_42,_ respectively) with time in the absence and presence of increasing quantities of 14-3-3ζ. The Aβ_42_ peptide is more hydrophobic than Aβ_40_ due to the presence of the additional Ile-Ala dipeptide at its C-terminus. Consistent with the observation by others [15], Aβ_42_ aggregates at a much faster rate (compare Figure 1a,b, noting the very different time scales), without the presence of a lag phase, compared to Aβ_40_ which has a very long lag phase of around 33 h under the conditions used. The protein 14-3-3ζ was much more effective at inhibiting the aggregation of Aβ_40_ than Aβ_42_. Thus, a 1.0:0.5 molar subunit ratio of Aβ_40_:14-3-3ζ completely inhibited the former’s aggregation (Figure 1a) whereas a two-molar excess of 14-3-3ζ only partially reduced fibril formation of Aβ_42_ (Figure 1b). In separate experiments, Aβ_40_ that had been seeded with Aβ_40_ fibrils aggregated much earlier, i.e., a lag phase of 12 h (Figure S1). An equimolar ratio of Aβ_40_:14-3-3ζ significantly delayed the onset of Aβ_40_ aggregation (lag phase of 29 h) and partially suppressed its extent of aggregation as monitored by ThT fluorescence. More pronounced effects on the lag phase and extent of Aβ_40_ fibril formation were observed at a 1.0:2.0 molar ratio, with complete inhibition of aggregation occurring at a 1.0:4.0 molar ratio of Aβ_40_:14-3-3ζ (Figure S1). 

Transmission electron microscopy (TEM) images confirmed the conclusions from the ThT data of Figure 1. In the absence of 14-3-3ζ, both Aβ peptides formed amyloid fibrils, with Aβ_40_ fibrils being longer than those of Aβ_42_ (compare Figure 1a,b). TEM images of Aβ_42_ showed agglomerates of fibrils alongside well-separated, distinct fibrillar species (Figure 1b). In the presence of 14-3-3ζ, the formation of amorphous-like aggregates was observed for both Aβ peptides, a phenomenon that commonly occurs when fibril-inhibiting molecules (large and small) interact with amyloid fibril-forming peptides and proteins (e.g., [16]). In summary, 14-3-3ζ inhibited the aggregation and fibril formation of Aβ_40_ at stoichiometric ratios. By contrast, 14-3-3ζ only partially inhibited the aggregation of Aβ_42_ at these ratios. 

Other studies have examined the in vitro chaperone ability of 14-3-3ζ against amorphously aggregating target proteins [7,8,10]. 14-3-3ζ is unlike sHsps as it is not highly promiscuous in its chaperone ability, i.e., 14-3-3ζ exhibits relatively selective chaperone ability against aggregating target proteins. However, like sHsps [17,18,19,20], 14-3-3ζ is a more efficient chaperone at inhibiting target proteins that are aggregating slowly and amorphously. Thus, target protein aggregation rate (i.e., kinetics) may be the major factor in determining the greater ability of 14-3-3ζ to inhibit the aggregation of Aβ_40_ more efficiently compared to the aggregation of Aβ_42_. The poorer efficiency of 14-3-3ζ at inhibiting the amyloid fibril formation of seeded Aβ_40_ (Figure S1), in which faster aggregation of the peptide occurred than for unseeded Aβ_40_ (Figure 1a), is consistent with this conclusion. Likewise, sHsps are more efficient at inhibiting slowly aggregating amyloid fibril-forming target proteins such as α-syn [12,21]. According to Mori et al. and Kollmer et al., Aβ_40_ is the major secreted form of the Aβ peptides in vivo and is the predominant species present in AD extracellular plaques [22,23]. The presence of 14-3-3 proteins extracellularly [11], where Aβ peptides are primarily located, implies that the ability of 14-3-3ζ to inhibit Aβ_40_ aggregation has physiological significance. Accordingly, the residue-specific interaction between Aβ_40_ and 14-3-3ζ was explored by solution-phase NMR spectroscopy.

**Figure 2:**
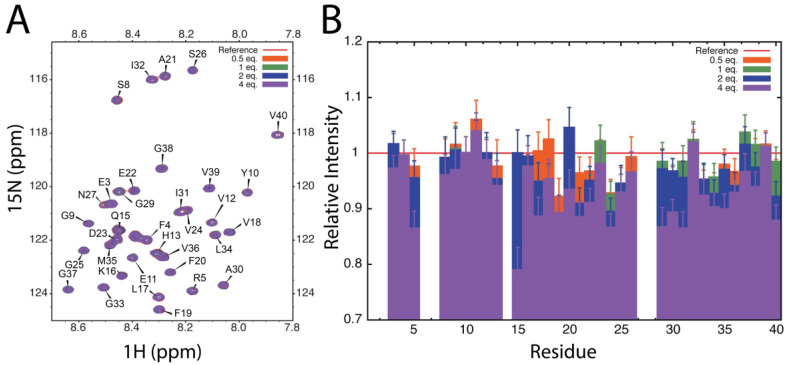
^1^H-^15^N HSQC NMR spectrum of uniformly ^15^N-labeled Aβ_40_ in the absence and presence of 14-3-3ζ. (**A**) Amide region of the ^1^H-^15^N HSQC spectrum at 5 °C of ^15^N-labeled Aβ_40_ in the presence of unlabeled 14-3-3ζ at 1.0:0.0–4.0 molar ratios of Aβ_40_:14-3-3ζ (red contour levels: 0.0, orange: 0.5, green: 1.0, blue: 2.0, and purple: 4.0 refer to the molar ratio of 14-3-3ζ to Aβ_40_). Assignments are from Hou and Zagorski [24]. No change in the ^1^H and ^15^N chemical shifts of Aβ_40_ occurred upon addition of 14-3-3ζ. (**B**) The change in the relative intensities of Aβ_40_ ^1^H-^15^N cross-peaks at 1.0:0.5-4.0 molar ratios of Aβ_40_:14-3-3ζ relative to Aβ_40_ alone (orange line). The intensities of the contour levels were corrected for dilution effects. Error bars represent the standard deviation in background noise of the spectra.

Figure 2a shows the ^1^H-^15^N HSQC spectrum of ^15^N-labeled Aβ_40_ at physiological pH and 5 °C, in the absence and presence of unlabeled 14-3-3ζ, at up to a four-molar excess of the chaperone protein. The NMR experiments were conducted at a low temperature to negate (or minimize) the possibility that Aβ_40_ would form amyloid fibrils during the timeframe of acquisition of the spectra. Thus, the NMR experiments monitored interactions between Aβ_40_ and 14-3-3ζ at the earliest stage of Aβ_40_ amyloid fibril formation.

Cross-peaks of Aβ_40_ are labeled in Figure 2a [24]. Cross-peaks were not observed for six of the 40 amino acids of Aβ_40_ (D1, A2, H6, D7, H14 and K28), presumably due to broadening associated with intermediate exchange. No change in chemical shifts of the observed cross-peaks occurred in the presence of 14-3-3ζ, implying weak and transient interactions and a relatively overall fast exchange between the two species. There was a non-uniform decrease in the intensity, or broadening, of the Aβ_40_ cross-peaks across its amino acid sequence in the presence of increasing concentrations of 14-3-3ζ compared to the spectrum of Aβ_40_ on its own (Figure 2b). The decrease in cross-peak intensity was most marked for Q15 to G25, encompassing the hydrophobic core of the peptide (L17-V18-F19-F20-A21), and most of the cross-peaks arising from the C-terminal region Aβ_40_ (G29 to V40), which is also hydrophobic in character. The NMR data imply that these regions interact with 14-3-3ζ during its chaperone function to inhibit Aβ_40_ amyloid fibril formation. As with the chaperone mechanism of the sHsp αB-crystallin in preventing amyloid fibril formation of Aβ_40_ [25], the interaction between the 14-3-3ζ and Aβ_40_ is transient in nature, which facilitates the potent stoichiometry of 14-3-3ζ in inhibiting Aβ_40_ aggregation. 

The recent determination of the structures of the amyloid fibrillar forms of Aβ peptides by cryo-electron microscopy (cryoEM) has provided an atomic-level description of the arrangement of the polypeptide backbone. Of most relevance is the cryoEM structure of Aβ_40_ fibrils isolated from the brain tissue of AD patients, post-mortem [23]. From this study, the four β-strands in the β-sheet fibril core of Aβ_40_ arise from residues A2-S8, Y10-H13, Q15-F19, and I32-L34. The latter two β-strands correspond well to the residues whose cross-peak intensities in the ^1^H-^15^N NMR spectrum of Aβ_40_ are decreased in the presence of 14-3-3ζ (Figure 2b). They also correspond well to the residues that form the cross β-sheet core of Aβ, as determined by solid state NMR spectroscopy [26,27]. Furthermore, of all the Aβ_40_ cross-peaks in Figure 2b, the intensity of the Q15 cross-peak was decreased the most (by over 20%) in the presence of a four-molar excess of 14-3-3ζ. Thus, 14-3-3ζ interacts preferentially with at least part of the Aβ_40_ peptide that forms its fibril core and, since it does so with Aβ_40_ amino acids within and nearby to Q15-F19 and I32-L34, it is surmised that these two β-strands are potentially the first to assemble during Aβ_40_ fibril formation. Furthermore, 14-3-3ζ inhibits Aβ_40_ fibril formation via interfering at the earliest stage of its aggregation pathway. Again, this mechanism has distinct parallels with that exhibited by sHsps during their prevention of amyloid fibril formation of a variety of proteins (summarized in [5,6,28]). Although Aβ-containing amyloid plaques are extracellular deposits, there is an intracellular component to Aβ aggregation prior to the peptide’s export into the extracellular medium [29]. Thus, in vivo interaction of Aβ with 14-3-3ζ intracellularly, along with the other 14-3-3 isoforms and sHsps, may have physiological importance in modulating Aβ aggregation and maintaining intracellular proteostasis. Furthermore, the extracellular presence of 14-3-3 proteins [11] would also provide the opportunity for their interaction with Aβ in this environment.

### 2.2. Interaction of 14-3-3ζ with α-Synuclein 

A53T α-syn is a mutant that aggregates at a faster rate compared to the WT protein and is associated with familial PD [30]. As monitored by ThT fluorescence, 14-3-3ζ had minimal ability to inhibit amyloid fibril formation of A53T α-syn at an equimolar ratio under physiological conditions of over 22 h of co-incubation (Figure S2). In agreement with this, Plotegher et al. [31] investigated the ability of all seven human 14-3-3 isoforms to inhibit α-syn fibril formation and found that two of the isoforms (η and τ (also termed θ)) were potent inhibitors of α-syn aggregation, whereas the others, including 14-3-3ζ, were ineffective. The inability of 14-3-3ζ to decrease A53T α-syn aggregation to any significant degree at a stoichiometric ratio may be due to the latter protein’s relatively rapid rate of aggregation, which commences around four hours of incubation compared to 33 h for the Aβ_40_ peptide (Figure 1a). The selective nature of 14-3-3ζ chaperone action against aggregating target proteins is also a factor since only the 14-3-3 η and τ/θ isoforms inhibit α-syn aggregation [31]. TEM images of A53T α-syn amyloid fibrils formed in the absence and presence of 14-3-3ζ were of very similar length and morphology, consistent with the inability of 14-3-3ζ to modify A53T α-syn fibril formation (Figure S2). Atomic force microscopic analysis of amyloid fibrils produced from incubated mixtures of α-syn and 14-3-3ζ also showed little difference in fibril morphology compared to that of mature α-syn amyloid fibrils [31].

**Figure 3:**
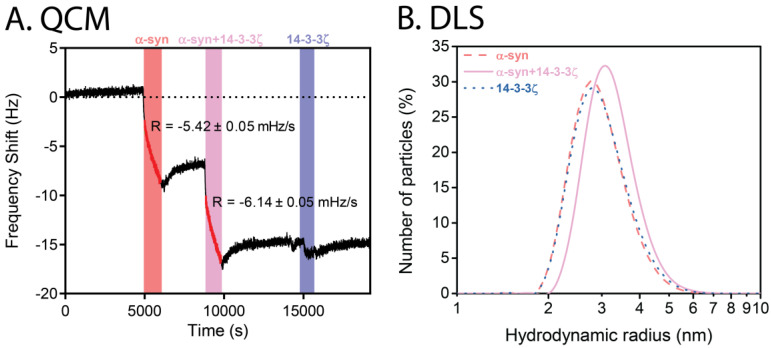
(**A**) QCM traces of the elongation of preformed WT α-syn amyloid fibrils in the presence and absence of 14-3-3ζ. Preformed amyloid fibrils were attached on to the chip and equilibrated with buffer for 24 h. At approximately 5000 s intervals, 10 μM WT α-syn monomer (red), 1.0:1.0 molar ratio of α-syn monomer:14-3-3ζ (pink), and 10 μM 14-3-3ζ (blue) were added. After the addition of each protein sample, the chip was washed with buffer until a plateau in signal was obtained (white). The rate of frequency change (R) is indicated for when α-syn monomer and a 1.0:1.0 molar ratio of α-syn:14-3-3ζ were washed over the QCM crystal. (**B**) DLS of WT α-syn and 14-3-3ζ. DLS profile and hydrodynamic radius of 20 μM WT α-syn at 25 °C in the absence (red dashed line) and presence (pink continuous line) of 14-3-3ζ at a 1.0:1.0 molar ratio. Also shown is the DLS profile of 20 μM 14-3-3ζ (blue dotted line).

A quartz crystal microbalance (QCM) measures the mass change of a surface upon the addition or removal of molecules as monitored by the change in frequency of a quartz crystal resonator. QCM was used to monitor the change in mass of WT α-syn preformed amyloid fibrils attached to the crystal upon flowing solutions of monomeric α-syn, an equimolar ratio of α-syn:14-3-3ζ and 14-3-3ζ itself over the crystal. The rate of change of frequency was essentially the same for the addition of α-syn in the absence or presence of 14-3-3ζ (Figure 3a), indicating that α-syn monomers bound to the α-syn fibrils on the crystal surface (thus increasing their size and mass), a process that was not affected by the presence of 14-3-3ζ. 14-3-3ζ did not bind to the α-syn fibrils. Thus, if any interaction between the two proteins occurs, it does so early along the aggregation pathway of α-syn, as with the interaction between α-syn and the sHsp, αB-crystallin [32,33]. The dynamic light scattering (DLS) profiles of WT α-syn and 14-3-3ζ were similar, with hydrodynamic radii at 37 °C and pH 7 of 2.789 ± 0.003 nm for WT α-syn and 2.818 ± 0.003 nm for 14-3-3ζ. The latter value compares well with the literature [8,34]. When both proteins were mixed together at an equimolar ratio, however, a single peak was observed at the larger hydrodynamic radius of 3.062 ± 0.001 nm (Figure 3b), implying some association between the two proteins under physiological conditions.

**Figure 4:**
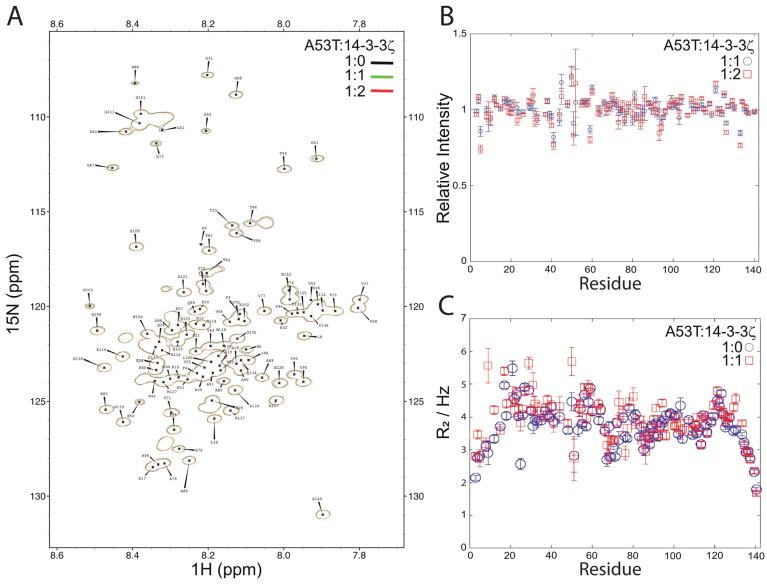
^1^H-^15^N HSQC NMR spectrum of uniformly ^15^N-labeled A53T α-syn in the absence and presence of 14-3-3ζ. (**A**) Amide region of the ^1^H-^15^N HSQC spectrum at 10 °C of ^15^N-labeled A53T α-syn (black) following the addition of one (green) and two (red) molar equivalents of unlabeled 14-3-3ζ. Assignments are from Dedmon et al. [35]. No change in the ^1^H and ^15^N chemical shifts of A53T α-syn occurred upon addition of 14-3-3ζ. (**B**) The change in the relative intensity of ^15^N-labeled A53T α-syn cross-peaks in the presence of 14-3-3ζ at 1.0:1.0 and 1.0:2.0 molar ratios, relative to the absence of 14-3-3ζ. The intensities of the contour levels were corrected for dilution effects. Three HSQC spectra were acquired for each sample and the data are plotted as the average ± SEM. (**C**) The spin-spin relaxation rate (R_2_) for individual backbone nitrogens of ^15^N-labeled A53T α-syn in the absence (blue) and presence (red), at a 1.0:1.0 molar ratio, of unlabeled 14-3-3ζ.

Accordingly, the interaction of α-syn and 14-3-3ζ was investigated by NMR spectroscopy at pH 7.4 and 10 °C using ^15^N-labeled A53T α-syn in the presence of unlabeled 14-3-3ζ at 1:1 and 1:2 molar ratios of A53T α-syn:14-3-3ζ. As with the interaction between Aβ_40_ and 14-3-3ζ, the NMR experiments were acquired at a low temperature to circumvent the possibility of fibril formation by A53T α-syn. Figure 4a shows the ^1^H-^15^N HSQC NMR spectrum of A53T α-syn under these conditions. The ^1^H NH chemical shifts are all contained within the ‘random coil’ chemical shift regime of 7.6 to 8.6 ppm, as expected since α-syn is a 140-amino acid intrinsically disordered protein of little or no stable secondary structure. In the presence of unlabeled 14-3-3ζ, no alteration in chemical shifts occurred of the α-syn cross-peaks. This was also observed for the ^1^H-^15^N HSQC NMR spectrum of a mixture of α-syn and 14-3-3η [31] and for α-syn interacting with αB-crystallin [32] and an unrelated molecular chaperone, Hsp70 [35]. The intensities of the cross-peaks in the HSQC spectra of A53T α-syn in the presence of 14-3-3ζ were measured, and their intensities relative to those in the spectrum of A53T α-syn only are plotted in Figure 4b. Alteration in cross-peak intensity was localized to the N-terminus (V3 to K10) and from V40 to K60, and possibly towards the C-terminus between D121 and D135. Together, these data imply that the interaction between A53T α-syn and 14-3-3ζ is weak and transient, with fast exchange. In their NMR studies of the interaction of α-syn with 14-3-3η, Plotegher et al. [31] did not examine the effects on the intensity of α-syn cross-peaks due to the presence of 14-3-3η. In contrast to the results presented herein with 14-3-3ζ, 14-3-3η was an effective inhibitor of α-syn amyloid fibril formation [31]. Overall, the results from both studies are consistent with a transient and weak interaction between the two proteins which, owing to some subtle structural and mechanistic differences between isoforms, leads to inhibition of α-syn oligomerization in the presence of 14-3-3η but not 14-3-3ζ.

The transverse (spin-spin) ^15^N relaxation rate (R_2_) of ^15^N-labeled A53T α-syn cross-peaks in the absence and presence of a molar equivalent of 14-3-3ζ was determined (Figure 4c). Interaction between the two proteins, even if transient, will alter (most likely increase) the R_2_ values of the residues involved. On average, there was a slight increase in R_2_ values for A53T α-syn in the presence of 14-3-3ζ which was concentrated in the first 50 or so amino acids, in agreement with the cross-peak intensity data in Figure 4b, i.e., comparison of R_2_ values in the absence and presence of 14-3-3ζ was consistent with an interaction between the two proteins that mainly involved the N-terminal region of A53T.

The preferential interaction of the N-terminal region of α-syn with 14-3-3ζ is consistent with the in vitro and in-cell NMR data of Burmann et al. [36]. ^1^H-^15^N HSQC spectra of ^15^N-labeled α-syn revealed that six molecular chaperones (they did not examine 14-3-3 proteins or sHsps) ‘commonly recognize a canonical motif in α-synuclein, consisting of the N-terminus (12 residues) and a segment (six residues) around Tyr39’. The interaction is transient and weak and, inside cells, maintains α-syn in its monomeric, unfolded, and functional (i.e., non-amyloidogenic) state. The two interacting N-terminal regions of α-syn are hydrophobic in nature, consistent with the primary role of hydrophobic interactions in the interaction of molecular chaperones. Mass spectrometric determination of the interactome of α-syn in mammalian cells revealed that many molecular chaperones are involved in interacting with α-syn via its N-terminal region, including 14-3-3ζ, along with three other 14-3-3 isoforms (ε, γ and θ/τ) [36]. Thus, during intracellular proteostasis, a diversity of molecular chaperones interacts with a common N-terminal interface of α-syn to prevent its association and amyloid fibril formation.

The association of α-syn and 14-3-3 proteins intra- and extracellularly has been demonstrated in other studies. Immunoprecipitation and immunoblotting of rat brain homogenates revealed co-association of α-syn and 14-3-3 proteins [37]. They also noted that α-syn and 14-3-3 proteins have significant sequence similarity in their N-terminal regions, i.e., of L8-E61 in α-syn and L44-S99 in 14-3-3ζ which would facilitate their mutual association. Wang et al. [38] determined from a cell biological study that the θ/τ isoform of 14-3-3 complexes to α-syn in a chaperone interaction, thereby preventing α-syn oligomerization and regulating the cell-to-cell transfer of cytotoxic α-syn. As a result, 14-3-3θ/τ reduces α-syn cell toxicity and, hence, may play an important role in the pathology associated with PD. 

Recently, Doherty et al. [39] identified that the N-terminal regions G36 to S42 and K45 to E57, particularly the former, are critical for regulating the aggregation of α-syn in vitro and in the nematode, *C. elegans*. These regions align well with those that interact with 14-3-3ζ and are obvious targets for the development of α-syn aggregation inhibitors. Furthermore, in the cryoEM-derived structure of the amyloid fibril form of a variant (residues 1 to 121) of α-syn without the last 19 amino acids of its acidic, proline-rich and unstructured C-terminal region, K43 to K58 is β-strand 3 that forms the interface between the two protofibrils in the overall fibrillar structure [40]. From cryoEM and solid-state NMR experiments on full-length fibrillar α-syn, a similar interface is present, but two different overall morphologies are formed compared to that for 1-121 α-syn, highlighting the polymorph nature of the protein in its amyloid fibrillar state [41]. Most of the first 37 residues of α-syn are not observed, presumably because they are disordered and mobile and therefore not part of the fibril core [40]. Thus, 14-3-3 proteins interacting transiently, in a chaperone manner, with K43 to K58 of A53T α-syn may preclude the formation of β-strand 3 (and its dimer association) in the earliest stages of α-syn aggregation. 

The cryoEM structures of α-syn fibrils (filaments) extracted from inclusions in the brains of individuals with multiple system atrophy (MSA) are also polymorphic. They comprise two types of fibrils that each contain two different protofibrils [42]. The MSA α-syn fibrils are different from those formed in vitro from recombinant α-syn in terms of the number and arrangement of β-strands. However, the MSA and recombinant α-syn structures contain a common interface between the protofibrils involving the N-terminal region of α-syn. For the MSA α-syn protofibrils, this encompasses Q24 to A56 (twice), G36 to V63 and L38 to T64. Again, these data are consistent with the role of the N-terminal region in regulating the self-association of α-syn and its interaction with molecular chaperones such as 14-3-3ζ.

## 3. Materials and Methods

*Reagents*. All reagents were of analytical grade and purchased from Sigma-Aldrich (Australia). Aβ peptides (1–40 and 1–42) were purchased from Bachem Ltd. (Weil am Rhein, Germany). In situ and ex situ Thioflavin T (ThT) fluorescence measurements were conducted in black, clear bottom 96 microwell plates (Greiner Bio-One, Baden-Württemberg, Germany) using SealPlate MiniStrips (Astral Scientific, Australia) to prevent evaporation. Uranyl acetate, used for negative staining of samples for TEM, was obtained from Agar Scientific (Essex, UK). All solutions were prepared using deionized water purified to a resistivity of 18.2 MΩ·cm and subsequently filtered through a 0.22 µm membrane (Millipore, Australia). 

*Protein expression and purification*. The 14-3-3ζ and Tobacco Etch Virus (TEV) protease plasmid constructs were a kind gift from Prof. James Murphy (Walter and Eliza Hall Institute of Medical Research, Australia) and Prof. Michael Parker (University of Melbourne, Australia), respectively. The expression plasmids for WT and A53T α-syn were a gift from Dr Tim Guilliams (University of Cambridge, UK). Recombinant 14-3-3ζ-His6 fusion proteins were expressed and purified as described previously [8,43,44]. Following purification, cleavage of the His6 tag was achieved using the TEV protease, and the cleavage products were purified using Ni-NTA column chromatography (Qiagen). WT and A53T α-syn were expressed and purified using the protocol of Narhi et al. [45]. ^15^N-labeled α-syn was prepared as outlined in Dedmon et al. [35]. Recombinant ^15^N-labeled Aβ peptides (1–40 and 1–42) were prepared by co-expression with an affibody [46]. The purified proteins were stored at −20 °C.

All protein and peptide concentrations were determined via absorbance measurements at either 276 nm or 280 nm using a Cary 5000 UV-vis spectrophotometer (Varian Ltd., Australia). A molar extinction coefficient (ε) of 5600 M^−1^ cm^−1^ was used for WT and A53T α-syn, measured at 276 nm. An ε of 23,860 M^−1^ cm^−1^ was used for 14-3-3ζ, measured at 280 nm.

*Dynamic Light Scattering of 14-3-3ζ and α-synuclein*. For 14-3-3ζ and α-syn alone or at a 1.0:1.0 molar ratio (7.2 μM) in 50 mM phosphate containing 100 mM NaCl and 2 mM EDTA, pH 7.4, time-resolved DLS analysis was performed at 37 °C using a Zetasizer Nano-ZS (Malvern Instruments, Worcestershire, UK). The particle diameter-intensity distribution and mean hydrodynamic diameter were determined from 13 acquired correllograms using the program CONTIN [47] and the method of cumulants [48], respectively, via Dispersion Technology Software (Malvern Instruments Ltd., Worcestershire, UK).

*Transmission Electron Microscopy imaging of amyloid fibrils formed in vitro*. An aliquot of the protein solutions from in situ ThT assays (6 μL) was transferred onto a carbon-coated nickel transmission electron microscopy (TEM) grid (SPI Supplies, West Chester, PA, USA). The grid was then washed using filtered MilliQ water (2 × 10 μL) before negative staining with uranyl acetate solution (8 μL, 2% *w*/*v*, in MilliQ). Between each step and after staining, excess solvent was removed by filter paper. After staining, the grids were left to air dry. Grids were viewed on a Philips CM 100 Transmission Electron Microscope (Eindhoven, Netherlands) between 13,500 and 64,000 times magnification operating at 120 kV.

*Chaperone assays to monitor the effect of 14-3-3ζ on amyloid fibril formation of amyloid β and α-synuclein*. All in vitro experiments in which amyloid fibrils were formed from Aβ_40_ and Aβ_42_ or A53T α-syn were undertaken at 37 °C in 50 mM phosphate, 100 mM NaCl, and pH 7.4. The formation of amyloid fibrils, in the absence or presence of 14-3-3ζ was assessed by ThT fluorescence (20 µM, excitation 440 nm, emission 490 nm) using a Fluorostar Optima plate reader (BMG Labtechnologies, Australia).

Aβ_40_ and Aβ_42_ were dissolved in ammonium hydroxide (final concentration 3.8 mM) and then diluted to 500 µM in water and stored at −80 °C. Further dilutions were made in 50 mM phosphate buffer containing 100 mM NaCl, pH 7.4, to achieve final concentrations for plate reader aggregation assays. The kinetics of Aβ_40_ and Aβ_42_ (15 µM, 100 µL) amyloid fibril formation, in the presence and absence of 14-3-3ζ, were monitored by the change in ThT fluorescence.

A53T α-syn and 14-3-3ζ solutions were prepared in 50 mM phosphate buffer containing 100 mM NaCl, pH 7.4. Each sample of either A53T α-syn (70 μM), 14-3-3ζ (70 μM), or A53T α-syn and 14-3-3ζ (70 μM of each protein) was separated into four Eppendorf tubes, each containing 500 μL. All samples were then wrapped in aluminium foil, with air-holes to aid in air circulation and temperature equilibration. They were incubated at 37 °C and shaken at 200 rpm for 24 h. For each protein solution, samples (2 × 5 µL) were taken every 2 h and added to 96 well plates (Greiner Bio-One, Baden-Wurttemberg, Germany) containing ThT (20 µM, 60 µL). The ThT fluorescence of each plate was read at 37 °C. Samples (8 µL) were also taken at 0, 4, 10, and 21 h for TEM imaging.

*Two-dimensional ^1^H-^15^N HSQC NMR spectroscopy of ^15^N-labeled Aβ_40_ and unlabeled 14-3-3ζ*. Samples of ^15^N-labeled Aβ_40_ and 14-3-3ζ were prepared separately in 20 mM phosphate containing 0.5 mM EDTA and 0.02 % *w*/*v* NaN_3_, pH 7.4. All spectra were recorded at 5 °C on a Bruker Avance 500 NMR spectrometer (Bruker, UK) operating at a magnetic field strength of 11.7 T and a ^1^H frequency of 500.1 MHz and a ^15^N frequency of 50.7 MHz equipped with ^1^H-^15^N, ^13^C, ^2^H z-gradient TCI cryoprobe. ^1^H chemical shifts were referenced to water as per Cavanagh et al. [49]. Data were processed with NMRPipe [50] and analyzed with Sparky v. 3.112 [51] software.

Gradient-enhanced two-dimensional ^1^H-^15^N heteronuclear single quantum coherence (HSQC) correlation spectra were acquired using water suppression via a Watergate pulse sequence. Spectra were acquired with 640 and 64 complex points and spectral widths of 5001.324 Hz and 532.180 Hz for the ^1^H and ^15^N dimensions, respectively. The carrier was set on-resonance with water in the ^1^H dimension and 16 scans were recorded per increment for 70 µM Aβ_40_. Up to four molar equivalences of 14-3-3ζ were then added in identical buffer, and subsequent NMR spectra were acquired with identical parameters. No chemical shift changes were observed in the HSQC spectra, and intensity changes were calculated (following correction for dilution), with error bars indicating the standard deviation in background noise of the spectra.

*Two-dimensional ^1^H-^15^N HSQC NMR spectroscopy of ^15^N-labeled A53T α-synuclein and unlabeled 14-3-3ζ*. All NMR spectra were acquired at 10 °C on a Bruker Avance III 700 NMR spectrometer operating at a magnetic field strength of 16.4 T and an ^1^H frequency of 700.1 MHz and a ^15^N frequency of 71.0 MHz equipped with a TXI cryoprobe. Data were processed with NMRPipe [50] and analyzed with Sparky v. 3.112 [51] software using previously reported assignments [35,52]. ^15^N A53T α-syn (100 µM) was dissolved in 50 mM sodium phosphate containing 100 µM NaCl (10% D_2_O, pH 7.4). DSS (4,4-dimethyl-4-silapentane-1-sulfonic acid, 0.1%) was used as a chemical shift reference [53]. Three sensitivity-enhanced ^1^H-^15^N HSQC spectra were acquired, with 256 increments, a sweep width of 25 ppm in the indirect dimension, and 4 scans per increment. 

^15^N spin-spin relaxation rates (R_2_) were determined for relaxation times of 15.8 and 126.7 ms with a Carr-Purcell-Meiboom-Gill (CPMG) frequency of 1 kHz, encoded as a ^1^H-^15^N HSQC with 128 increments and 16 scans per increment, with a recycle delay of 2 s. One equivalent of 14-3-3ζ was then added in identical buffer, resulting in final α-syn and 14-3-3ζ concentrations of 84 µM, and a second set of NMR spectra were acquired with identical parameters. No chemical shift changes were observed in the HSQC spectra, and intensity changes were calculated (following correction for dilution) from the mean of the three spectra, with error bars indicating the standard error of the mean. All A53T α-syn cross-peak positions were obtained from an independent HSQC experiment to avoid the introduction of systematic bias that results from coupled measurements of peak position and intensity.

*Quartz Crystal Microbalance of α-synuclein and 14-3-3ζ interaction*. QCM experiments were performed as outlined in Shammas et al. [54].

## 4. Conclusions

Solution-phase NMR spectroscopy has provided detailed characterization of the regions or interfaces of two disease-related, amyloid fibril-forming proteins, Aβ_40_ and α-syn, that interact with the molecular chaperone, 14-3-3ζ. For both Aβ_40_ and α-syn, the hydrophobic regions that interact with 14-3-3ζ are integral components of the β-sheet core within the final amyloid fibrillar structures of Aβ_40_ and α-syn prepared in vitro, and in vivo samples isolated from diseased individuals. The implication is that 14-3-3ζ interacts preferentially with these β-strand-forming regions of Aβ_40_ and α-syn early along their aggregation pathway and thereby interferes with their propensity to associate and form a β-sheet as the first step in amyloid fibril formation. In the case of α-syn, the N-terminal V40 to K60 region that interacts with 14-3-3ζ mainly encompasses the regions that regulate the protein’s aggregation [39].

As with sHsp chaperone action, intimate details of the mechanism by which 14-3-3 proteins elicit their chaperone action are not known, and neither are the reasons for the variation in their ability to inhibit the amorphous and fibrillar aggregation of destabilized peptides and proteins [7,8,10,31]. The chaperone action of 14-3-3ζ probably arises from at least partial dissociation of the dimer and exposure of the chaperone interaction site(s) at the dimer interface [44]. The significant variation in 14-3-3ζ chaperone ability (in this case, fibril formation of the Aβ peptides and A53T α-syn) is due to a combination of factors that have parallels with the chaperone action of the ten human sHsps [5,6]: (i) slowly aggregating target peptides or proteins provide the opportunity for 14-3-3ζ to interact efficiently, for example, via dissociation and exposure of its chaperone binding site(s); (ii) the nature of the partially folded intermediate state of the target peptide or protein prior to it forming the prefibrillar aggregate; (iii) the size/dimensions of the target peptide or protein. The seven 14-3-3 isoforms have different functional roles in vivo, most of which have not been elucidated. For example, the θ/τ isoform inhibits α-syn oligomerization and fibril formation [31,38], whereas the other isoforms, apart from η, are ineffective [31]. Furthermore, 14-3-3θ/τ reduces cell-to-cell transfer of α-syn, as occurs in the pathology of PD [38]. Similar diverse functionality, and some redundancy, occur within sHsps [5,6].

In conclusion, this study has provided insights into the means by which 14-3-3ζ (and, presumably, other 14-3-3 isoforms) exerts its chaperone action to inhibit amyloid fibril formation. The conclusions are generally applicable as there are strong parallels between the results reported herein and the in vitro and in-cell NMR studies of the interaction of α-syn with other molecular chaperones [36].

## Data Availability

The data presented in this study are available on request from the corresponding author.

## Supplementary Materials

The following are available online. Figure S1: Amyloid fibril formation, as monitored by ThT fluorescence, of seeded Aβ_40_ (15 μM) in the absence and presence of 14-3-3ζ at 1.0:1.0–4.0 molar ratios of Aβ_40_:14-3-3ζ, Figure S2: Amyloid fibril formation of A53T α-syn (70 μM) in the absence of 14-3-3ζ and at a 1.0:1.0 molar ratio of A53T α-syn:14-3-3ζ, as monitored by ThT fluorescence and TEM.

## Sample Availability

Samples of the proteins are not available from the authors.

## References

[1] Chiti F., Dobson C.M. (2006). Protein misfolding, functional amyloid, and human disease. Annu. Rev. Biochem..

[2] Knowles T.P., Vendruscolo M., Dobson C.M. (2014). The amyloid state and its association with protein misfolding diseases. Nat. Rev. Mol. Cell Biol..

[3] Powers E.T., Morimoto R.I., Dillin A., Kelly J.W., Balch W.E. (2009). Biological and chemical approaches to diseases of proteostasis deficiency. Annu. Rev. Biochem..

[4] Walther D.M., Kasturi P., Zheng M., Pinkert S., Vecchi G., Ciryam P., Morimoto R.I., Dobson C.M., Vendruscolo M., Mann M. (2015). Widespread proteome remodeling and aggregation in aging *C. elegans*. Cell.

[5] Treweek T.M., Meehan S., Ecroyd H., Carver J.A. (2015). Small heat-shock proteins: Important players in regulating cellular proteostasis. Cell. Mol. Life Sci..

[6] Hayashi J., Carver J.A. (2020). The multifaceted nature of alphaB-crystallin. Cell Stress Chaperones.

[7] Yano M., Nakamuta S., Wu X., Okumura Y., Kido H. (2006). A novel function of 14-3-3 protein: 14-3-3zeta is a heat-shock-related molecular chaperone that dissolves thermal-aggregated proteins. Mol. Biol. Cell.

[8] Williams D.M., Ecroyd H., Goodwin K.L., Dai H., Fu H., Woodcock J.M., Zhang L., Carver J.A. (2011). NMR spectroscopy of 14-3-3zeta reveals a flexible C-terminal extension: Differentiation of the chaperone and phosphoserine-binding activities of 14-3-3zeta. Biochem. J..

[9] Pair F.S., Yacoubian T.A. (2021). 14-3-3 proteins: Novel pharmacological targets in neurodegenerative diseases. Trends Pharmacol. Sci..

[10] Sluchanko N.N., Gusev N.B. (2017). Moonlighting chaperone-like activity of the universal regulatory 14-3-3 proteins. FEBS J..

[11] Kaplan A., Bueno M., Fournier A.E. (2017). Extracellular functions of 14-3-3 adaptor proteins. Cell Signal..

[12] Cox D., Carver J.A., Ecroyd H. (2014). Preventing alpha-synuclein aggregation: The role of the small heat-shock molecular chaperone proteins. Biochim. Biophys. Acta.

[13] Foote M., Zhou Y. (2012). 14-3-3 proteins in neurological disorders. Int. J. Biochem. Mol. Biol..

[14] LeVine H. (1999). Quantification of beta-sheet amyloid fibril structures with thioflavin T. Methods Enzymool..

[15] Jarrett J.T., Berger E.P., Lansbury P.T. (1993). The carboxy terminus of the beta amyloid protein is critical for the seeding of amyloid formation: Implications for the pathogenesis of Alzheimer’s disease. Biochemistry.

[16] Hudson S.A., Ecroyd H., Dehle F.C., Musgrave I.F., Carver J.A. (2009). (−)-Epigallocatechin-3-gallate (EGCG) maintains kappa-casein in its pre-fibrillar state without redirecting its aggregation pathway. J. Mol. Biol..

[17] Lindner R.A., Treweek T.M., Carver J.A. (2001). The molecular chaperone alpha-crystallin is in kinetic competition with aggregation to stabilize a monomeric molten-globule form of alpha-lactalbumin. Biochem. J..

[18] Carver J.A., Lindner R.A., Lyon C., Canet D., Hernandez H., Dobson C.M., Redfield C. (2002). The interaction of the molecular chaperone alpha-crystallin with unfolding alpha-lactalbumin: A structural and kinetic spectroscopic study. J. Mol. Biol..

[19] Treweek T.M., Rekas A., Lindner R.A., Walker M.J., Aquilina J.A., Robinson C.V., Horwitz J., Perng M.D., Quinlan R.A., Carver J.A. (2005). R120G alphaB-crystallin promotes the unfolding of reduced alpha-lactalbumin and is inherently unstable. FEBS J..

[20] Kulig M., Ecroyd H. (2012). The small heat-shock protein alphaB-crystallin uses different mechanisms of chaperone action to prevent the amorphous versus fibrillar aggregation of alpha-lactalbumin. Biochem. J..

[21] Cox D., Selig E., Griffin M.D., Carver J.A., Ecroyd H. (2016). Small heat-shock proteins prevent alpha-synuclein aggregation via transient interactions and their efficacy is affected by the rate of aggregation. J. Biol. Chem..

[22] Mori H., Takio K., Ogawara M., Selkoe D.J. (1992). Mass spectrometry of purified amyloid beta protein in Alzheimer’s disease. J. Biol. Chem..

[23] Kollmer M., Close W., Funk L., Rasmussen J., Bsoul A., Schierhorn A., Schmidt M., Sigurdson C.J., Jucker M., Fandrich M. (2019). Cryo-EM structure and polymorphism of Abeta amyloid fibrils purified from Alzheimer’s brain tissue. Nat. Commun..

[24] Hou L., Zagorski M.G. (2006). NMR reveals anomalous copper(II) binding to the amyloid Abeta peptide of Alzheimer’s disease. J. Am. Chem. Soc..

[25] Narayan P., Meehan S., Carver J.A., Wilson M.R., Dobson C.M., Klenerman D. (2012). Amyloid-beta oligomers are sequestered by both intracellular and extracellular chaperones. Biochemistry.

[26] Petkova A.T., Ishii Y., Balbach J.J., Antzutkin O.N., Leapman R.D., Delaglio F., Tycko R. (2002). A structural model for Alzheimer’s beta-amyloid fibrils based on experimental constraints from solid state NMR. Proc. Natl. Acad. Sci. USA.

[27] Paravastu A.K., Qahwash I., Leapman R.D., Meredith S.C., Tycko R. (2009). Seeded growth of beta-amyloid fibrils from Alzheimer’s brain-derived fibrils produces a distinct fibril structure. Proc. Natl. Acad. Sci. USA.

[28] Carver J.A., Ecroyd H., Truscott R.J.W., Thorn D.C., Holt C. (2018). Proteostasis and the regulation of intra- and extracellular protein aggregation by ATP-independent molecular chaperones: Lens alpha-crystallins and milk caseins. Acc. Chem. Res..

[29] Friedrich R.P., Tepper K., Ronicke R., Soom M., Westermann M., Reymann K., Kaether C., Fandrich M. (2010). Mechanism of amyloid plaque formation suggests an intracellular basis of Abeta pathogenicity. Proc. Natl. Acad. Sci. USA.

[30] Conway K.A., Harper J.D., Lansbury P.T. (1998). Accelerated in vitro fibril formation by a mutant alpha-synuclein linked to early-onset Parkinson disease. Nat. Med..

[31] Plotegher N., Kumar D., Tessari I., Brucale M., Munari F., Tosatto L., Belluzzi E., Greggio E., Bisaglia M., Capaldi S. (2014). The chaperone-like protein 14-3-3eta interacts with human alpha-synuclein aggregation intermediates rerouting the amyloidogenic pathway and reducing alpha-synuclein cellular toxicity. Hum. Mol. Genet..

[32] Rekas A., Adda C.G., Aquilina J.A., Barnham K.J., Sunde M., Galatis D., Williamson N.A., Masters C.L., Anders R.F., Robinson C.V. (2004). Interaction of the molecular chaperone alphaB-crystallin with alpha-synuclein: Effects on amyloid fibril formation and chaperone activity. J. Mol. Biol..

[33] Rekas A., Jankova L., Thorn D.C., Cappai R., Carver J.A. (2007). Monitoring the prevention of amyloid fibril formation by alpha-crystallin. Temperature dependence and the nature of the aggregating species. FEBS J..

[34] Sluchanko N.N., Tugaeva K.V., Greive S.J., Antson A.A. (2017). Chimeric 14-3-3 proteins for unraveling interactions with intrinsically disordered partners. Sci. Rep..

[35] Dedmon M.M., Christodoulou J., Wilson M.R., Dobson C.M. (2005). Heat shock protein 70 inhibits alpha-synuclein fibril formation via preferential binding to prefibrillar species. J. Biol. Chem..

[36] Burmann B.M., Gerez J.A., Matecko-Burmann I., Campioni S., Kumari P., Ghosh D., Mazur A., Aspholm E.E., Sulskis D., Wawrzyniuk M. (2020). Regulation of alpha-synuclein by chaperones in mammalian cells. Nature.

[37] Ostrerova N., Petrucelli L., Farrer M., Mehta N., Choi P., Hardy J., Wolozin B. (1999). Alpha-synuclein shares physical and functional homology with 14-3-3 proteins. J. Neurosci..

[38] Wang B., Underwood R., Kamath A., Britain C., McFerrin M.B., McLean P.J., Volpicelli-Daley L.A., Whitaker R.H., Placzek W.J., Becker K. (2018). 14-3-3 proteins reduce cell-to-cell transfer and propagation of pathogenic alpha-synuclein. J. Neurosci..

[39] Doherty C.P.A., Ulamec S.M., Maya-Martinez R., Good S.C., Makepeace J., Khan G.N., van Oosten-Hawle P., Radford S.E., Brockwell D.J. (2020). A short motif in the N-terminal region of alpha-synuclein is critical for both aggregation and function. Nat. Struct. Mol. Biol..

[40] Guerrero-Ferreira R., Taylor N.M., Mona D., Ringler P., Lauer M.E., Riek R., Britschgi M., Stahlberg H. (2018). Cryo-EM structure of alpha-synuclein fibrils. Elife.

[41] Guerrero-Ferreira R., Taylor N.M., Arteni A.A., Kumari P., Mona D., Ringler P., Britschgi M., Lauer M.E., Makky A., Verasdonck J. (2019). Two new polymorphic structures of human full-length alpha-synuclein fibrils solved by cryo-electron microscopy. Elife.

[42] Schweighauser M., Shi Y., Tarutani A., Kametani F., Murzin A.G., Ghetti B., Matsubara T., Tomita T., Ando T., Hasegawa K. (2020). Structures of alpha-synuclein filaments from multiple system atrophy. Nature.

[43] Woodcock J.M., Coolen C., Goodwin K.L., Baek D.J., Bittman R., Samuel M.S., Pitson S.M., Lopez A.F. (2015). Destabilisation of dimeric 14-3-3 proteins as a novel approach to anti-cancer therapeutics. Oncotarget.

[44] Woodcock J.M., Goodwin K.L., Sandow J.J., Coolen C., Perugini M.A., Webb A.I., Pitson S.M., Lopez A.F., Carver J.A. (2018). Role of salt bridges in the dimer interface of 14-3-3zeta in dimer dynamics, N-terminal alpha-helical order, and molecular chaperone activity. J. Biol. Chem..

[45] Narhi L., Wood S.J., Steavenson S., Jiang Y., Wu G.M., Anafi D., Kaufman S.A., Martin F., Sitney K., Denis P. (1999). Both familial Parkinson’s disease mutations accelerate alpha-synuclein aggregation. J. Biol. Chem..

[46] Macao B., Hoyer W., Sandberg A., Brorsson A.C., Dobson C.M., Hard T. (2008). Recombinant amyloid beta-peptide production by coexpression with an affibody ligand. BMC Biotechnol..

[47] Provencher S.W. (1982). CONTIN: A general purpose constrained regularization program for inverting noisy linear algebraic and integral equations. Comput. Phys. Commun..

[48] Koppel D.E. (1972). Analysis of macromolecular polydispersity in intensity correlation spectroscopy: The method of cumulants. J. Chem. Phys..

[49] Cavanagh J., Fairbrother W.J., Palmer A.G., Rance M., Skelton N.J., Cavanagh J., Fairbrother W.J., Palmer A.G., Rance M., Skelton N.J. (2007). Heteronuclear NMR experiments. Protein NMR Spectroscopy.

[50] Delaglio F., Grzesiek S., Vuister G.W., Zhu G., Pfeifer J., Bax A. (1995). NMRPipe: A multidimensional spectral processing system based on UNIX pipes. J. Biomol. NMR.

[51] Goddard T.D., Kneller D.G. (2008). SPARKY 3.

[52] Bussell R., Eliezer D. (2001). Residual structure and dynamics in Parkinson’s disease-associated mutants of alpha-synuclein. J. Biol. Chem..

[53] Wishart D.S., Bigam C.G., Holm A., Hodges R.S., Sykes B.D. (1995). 1H, 13C and 15N random coil NMR chemical shifts of the common amino acids. I. Investigations of nearest-neighbor effects. J. Biomol. NMR.

[54] Shammas S.L., Waudby C.A., Wang S., Buell A.K., Knowles T.P., Ecroyd H., Welland M.E., Carver J.A., Dobson C.M., Meehan S. (2011). Binding of the molecular chaperone alphaB-crystallin to Abeta amyloid fibrils inhibits fibril elongation. Biophys. J..

