# Supplementary material for: The Amyloid Fibril-Forming β-Sheet Regions of Amyloid β and α-Synuclein Preferentially Interact with the Molecular Chaperone 14-3-3ζ
Source: Molecules. 2021 Oct 11;26(20):6120. doi: 10.3390/molecules26206120 (PMC8538830; doi:10.3390/molecules26206120)
Supplement: Supplementary file 1 [file molecules-26-06120-s001.zip › molecules-1364882-supplementary.pdf]

Supplementary data

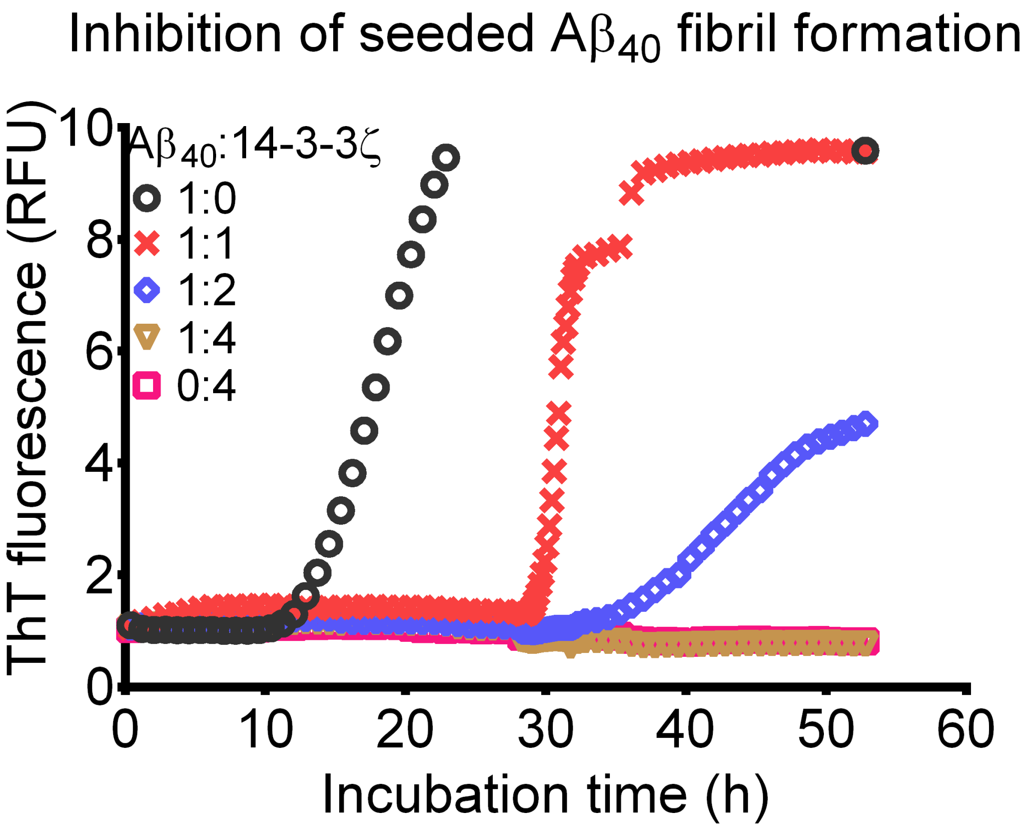

Figure S1. Amyloid fibril formation, as monitored by ThT fluorescence, of seeded Aβ_{40} (15 μM) in the absence and presence of 14-3-3ζ at 1.0:1.0-4.0 molar ratios of Aβ_{40}:14-3-3ζ.

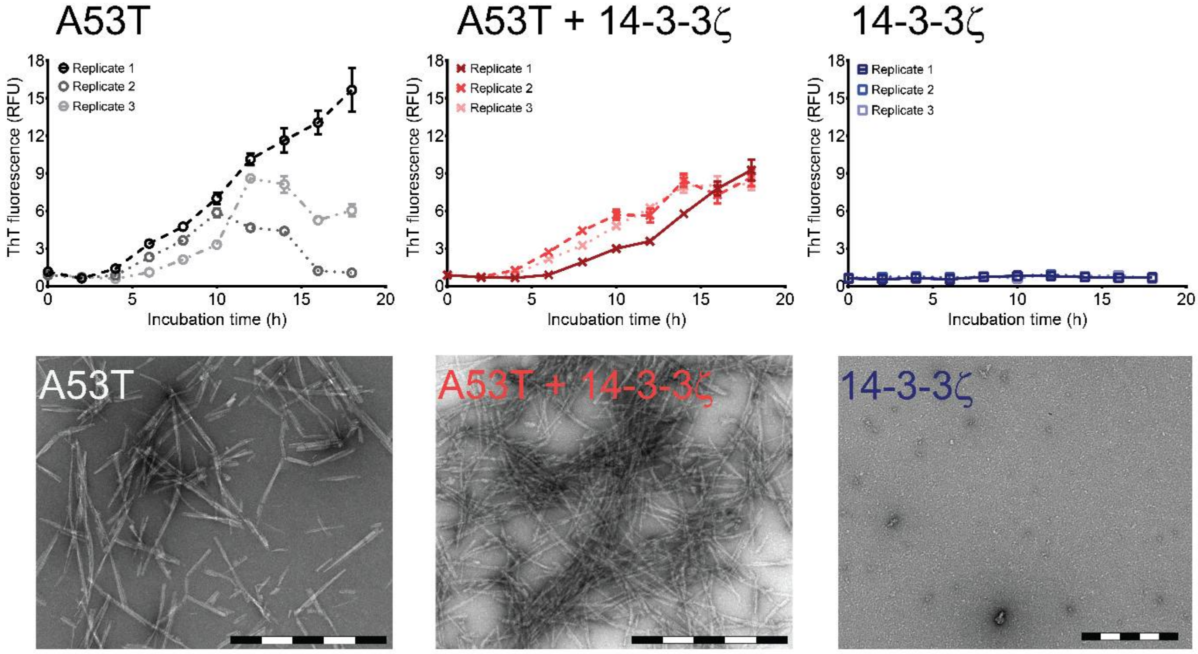

Figure S2. Top: Amyloid fibril formation, as monitored by ThT fluorescence, of A53T α-syn (70 μM) in the absence of 14-3-3ζ and at a 1.0:1.0 molar ratio of A53T α-syn: 14-3-3ζ. The decrease in ThT fluorescence for A53T α-syn on its own after ~ 12 hours of incubation most likely arises from precipitation of the fibrillar protein. The error bars represent the standard deviation of three technical replicates. Below: TEM images of these samples after incubation for 22 hours at 37 °C. Also shown on the right are the time-dependent ThT data for 14-3-3ζ on its own and a TEM image of 14-3-3ζ after incubation for 22 hours at 37 °C which provide no evidence of large-scale aggregation and fibril formation of 14-3-3ζ. Scale bars in all TEM images represent 1 μm.
